# Finite Element Analysis of a PTFE Felt Interposition Pad for Relieving Vascular Compression of the Trigeminal Nerve

**DOI:** 10.1155/abb/1115811

**Published:** 2026-09-24

**Authors:** Fang Wang, Zhong Yang, Yan Lv, Dan Jing, Min He

**Affiliations:** ^1^ Department of Neurosurgery, Nanjing Drum Tower Hospital, Affiliated Hospital of Medical School, Nanjing University, Nanjing 210008, China, nju.edu.cn; ^2^ School of Mechanical Engineering, Yancheng Institute of Technology, Yancheng 224001, China, ycit.edu.cn

**Keywords:** compressive stress, FEM, microvascular decompression, PTFE felt pad, trigeminal neuralgia

## Abstract

Vascular compression of the trigeminal nerve is considered a major pathological factor in trigeminal neuralgia, a debilitating facial pain disorder. This study presents a contact mechanics analysis of the interaction between the blood vessels, particularly the superior cerebellar artery, and the trigeminal nerve. Using finite element method (FEM), the mechanical stresses acting on the nerve at the root entry zone, a common site of neurovascular compression, were investigated. The results indicate that vascular contact generates significant local compressive stress on the trigeminal nerve. Further FEM analysis predicts that polytetrafluoroethylene (PTFE) felt interposition substantially reduces contact compressive stress. Under the material properties and loading conditions adopted in the present model, the compressive stress acting on the trigeminal nerve was reduced by ~95%. Sensitivity analyses demonstrated that the qualitative conclusion of substantial stress reduction remained unchanged over a plausible range of nerve elastic moduli and contact friction coefficients. The proposed model provides a biomechanical framework for understanding neurovascular compression and the mechanical effects of PTFE felt interposition. In addition, the model may serve as a useful tool for evaluating the influence of interposition material properties and optimizing decompression strategies in microvascular decompression (MVD) surgery.

## 1. Introduction

Recent clinical studies have further confirmed the effectiveness of microvascular decompression (MVD) in treating cranial nerve compression disorders. Long‐term clinical investigations have demonstrated that MVD can achieve high success rates in relieving symptoms caused by vascular compression of cranial nerves. For example, Zuo Huancong et al. [1] reported a large clinical study in China involving more than 4000 cases, showing that improvements in surgical techniques significantly enhanced the effectiveness of MVD, with a total success rate of 97%. These findings highlight the important role of MVD in the treatment of neurovascular compression syndromes.

Despite the clinical success of MVD, the exact etiology and pathogenesis of trigeminal neuralgia remain incompletely understood. A widely accepted theory suggests that chronic vascular compression leads to focal demyelination of the trigeminal nerve, resulting in abnormal neural transmission and severe facial pain. However, the mechanisms through which vascular compression causes nerve dysfunction remain complex and multifactorial. Therefore, quantitative investigation of vascular–nerve interactions requires a biomechanical perspective.

The mechanical behavior of blood vessels is largely determined by the structure and composition of the vascular wall. The extracellular matrix and connective tissue components play essential roles in maintaining vascular mechanical integrity and physiological function [2–4]. In particular, elastin, collagen, and vascular smooth muscle cells contribute significantly to arterial stiffness and elasticity [5–7]. Arterial stiffness has also been extensively investigated in the context of cardiovascular diseases and hypertension [8–10], and recent studies have shown that increased stiffness of vascular smooth muscle cells can significantly influence arterial mechanical behavior [11].

To quantify vascular mechanical properties, various experimental techniques have been developed to measure the elastic modulus of vascular tissues. Matrix stiffness has been shown to strongly affect vascular smooth muscle cell phenotype and function [12]. Atomic force microscopy has been widely used to characterize the mechanical properties of vascular tissues and atherosclerotic plaques [13, 14], while other studies have applied AFM‐based techniques to investigate the mechanical characteristics of arterial walls and endothelial tissues [15–17]. In addition, several theoretical models have been proposed to describe arterial stress–strain relationships and the mechanical responses of vascular smooth muscle [18–20].

In parallel with experimental studies, computational modeling—particularly the finite element method (FEM)—has become an important tool for analyzing vascular deformation and biomechanical interactions. Early studies introduced FEM‐based approaches for modeling soft tissue deformation in virtual surgery simulations [21]. Particle–spring and other computational models have subsequently been developed to improve the simulation accuracy of vascular wall deformation [22, 23]. Additional studies have investigated vessel deformation in surgical simulations and medical training systems [24, 25]. Anatomical studies have also highlighted the importance of understanding the spatial relationships between cranial nerves and surrounding vessels in the cerebellopontine angle during MVD surgery [26]. Further developments in vascular modeling have enabled improved geometric reconstruction and deformation simulation for surgical applications [27–29], while more recent work has addressed nonlinear deformation behavior and force feedback modeling in vascular intervention simulations [30, 31].

Recent clinical studies have also confirmed the effectiveness of modern MVD techniques. Peng et al. [32] reported favorable outcomes using fully endoscopic MVD for treating trigeminal neuralgia and related cranial nerve disorders. Similarly, Castelli et al. [33] demonstrated the long‐term reliability and low recurrence rate of MVD in a large cohort of trigeminal neuralgia patients.

Although MVD has achieved remarkable clinical success, the mechanical interaction between the compressing blood vessel and the trigeminal nerve remains insufficiently understood. Most previous studies have primarily focused on clinical outcomes and surgical techniques, while the biomechanical mechanisms underlying vascular compression and decompression have received relatively limited attention. According to the classical biomechanical theory of living tissues proposed by Fung [34], biological soft tissues exhibit nonlinear mechanical behavior. Therefore, quantitative biomechanical analysis is necessary to better understand the contact mechanics between blood vessels and the trigeminal nerve during compression.

In clinical practice, several surgical strategies are employed in MVD, including interposition, transposition, and their combination. The present study focuses specifically on the interposition technique, in which a polytetrafluoroethylene (PTFE) felt pad is placed between the compressing vessel and the trigeminal nerve. Although transposition may provide more effective decompression in certain cases, interposition remains a widely used surgical approach. Therefore, the biomechanical analysis presented in this study is limited to this specific surgical configuration.

Based on this framework, the finite element method (FEM) was employed to investigate the mechanical interaction between the trigeminal nerve and the compressing vessel. A biomechanical model incorporating the trigeminal nerve, the offending vessel, and a PTFE felt pad was established to simulate the decompression process during MVD. By comparing the stress distributions before and after PTFE felt interposition, the mechanical effects of neurovascular decompression were evaluated. The findings provide a biomechanical basis for understanding the role of interposition materials in reducing neurovascular compression and may serve as a useful tool for optimizing decompression strategies and material properties in future studies.

## 2. MVD Surgery

**Figure 1:**
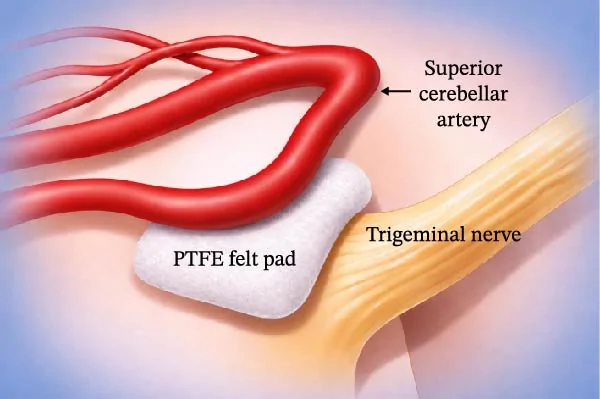
Schematic illustration of the MVD procedure showing PTFE felt interposition between the compressing vessel and the trigeminal nerve.

In MVD surgery for trigeminal neuralgia caused by vascular compression, a critical step involves identifying the neurovascular conflict under microscopic visualization. Decompression is then achieved by separating the compressing vessel from the trigeminal nerve and placing a PTFE felt pad to maintain the separation, as illustrated in Figure 1.

**Figure 2:**
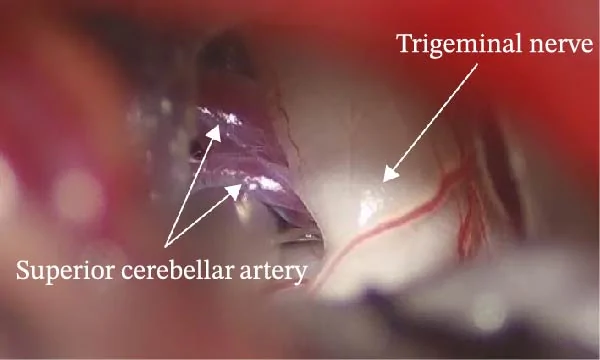
Intraoperative photograph showing the superior cerebellar artery compressing the trigeminal nerve before PTFE felt interposition during MVD surgery.

The case presented here was treated at Nanjing Drum Tower Hospital and is included solely to illustrate the surgical context of PTFE felt interposition. Preoperative imaging, shown in Figure 2, revealed that the superior cerebellar artery was in close contact with and compressing the root entry zone of the trigeminal nerve.

**Figure 3:**
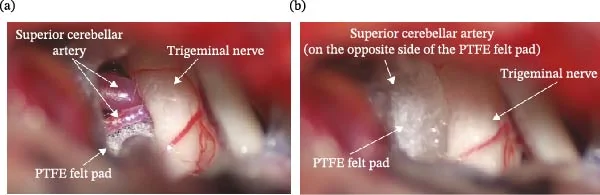
Intraoperative photograph showing the PTFE felt pad positioned between the compressing vessel and the trigeminal nerve during MVD surgery. (a) PTFE felt pad being placed. (b) PTFE felt pad placed between the artery and the nerve.

During surgery, the neurovascular relationship was visualized under an operating microscope. The compressing vessel was carefully dissected away from the trigeminal nerve, and a PTFE felt pad was positioned between the vessel and the nerve to maintain decompression, as shown in Figure 3. Postoperative imaging demonstrated successful neurovascular separation at the time of follow‐up, although future displacement, adhesion, granuloma formation, or recurrent compression cannot be completely excluded.

This case is presented only as an illustrative example of the surgical procedure and should not be interpreted as clinical validation of the finite element model. No standardized pain assessment, long‐term follow‐up, recurrence analysis, or complication evaluation were performed as part of the present study.

## 3. Theoretical Model

The decompression effect of the PTFE felt pad in the aforementioned MVD surgery was analyzed using the finite element method. The finite element method (FEM) is a numerical technique widely used to solve complex structural and biomechanical problems, particularly when analytical solutions are difficult or impossible. In this study, FEM is employed to model the mechanical interaction between the trigeminal nerve and a compressing blood vessel, most commonly the superior cerebellar artery, in the context of trigeminal neuralgia. The goal is to simulate and analyze the compressive stress distribution on the trigeminal nerve due to vascular compression and evaluate the mechanical effectiveness of MVD therapy.

### 3.1. Material Properties

The material properties of the nerve, blood vessel, and PTFE felt pad are critical for accurate simulation. These characteristics are based on experiments and equivalent assumptions. Blood vessels are a type of soft tissue and share common mechanical characteristics with other soft tissues. Fung [34] pointed out that soft tissues like blood vessels exhibit biomechanical properties such as heterogeneity, anisotropy, nonlinearity, residual stress, and near‐incompressibility. Blood vessels are complex, living composite materials and are classified as viscoelastic bodies. Their rheological properties are highly complex. The constitutive relationship of blood vessels describes their material properties under specific conditions, and when the conditions change, the constitutive relationship also changes. Therefore, there is currently no constitutive relationship that can accurately describe all the mechanical properties of blood vessels. When studying specific problems, it is essential to understand which properties of blood vessels are relevant to the problem being investigated to simplify the model.

This article primarily investigates the contact mechanics between blood vessels and nerves, with relatively low sensitivity to the nonlinear characteristics of material constitutive relationships. Hence, it is assumed that these materials behave as linear elastic bodies, and their modulus is measured through conventional tensile tests. PTFE felt pad is a highly flexible material with a much smaller elastic modulus; its elastic modulus is challenging to measure using traditional tensile and compressive experiments. In this study, it is determined through equivalent experimental methods.

In this study, porcine cardiovascular tissues were used for elastic modulus testing. The vessel selected was the porcine thoracic aorta, a large elastic artery connected to the heart and located at the superior portion of the heart within the thoracic cavity. The aorta was obtained from freshly slaughtered pigs at a local slaughterhouse. After harvesting, the surrounding connective tissues were carefully removed, and the vessel was rinsed with physiological saline prior to mechanical testing. Since the tissues were collected from animals slaughtered for food production and no animals were sacrificed specifically for this study, ethical approval was not required according to the institutional guidelines. The porcine thoracic aorta has a multilayered structure rich in elastic and collagen fibers, which provides good elasticity and pronounced nonlinear mechanical behavior. Although its structure is not identical to that of cerebral arteries, the porcine aorta is widely used as a representative arterial tissue in biomechanical studies due to its availability and comparable mechanical properties.

Due to the difficulties associated with experimentally measuring the elastic modulus of the nerve tissue, the elastic modulus of the nerve was assumed to be comparable to that of the blood vessel in the present study. Moreover, the primary objective of this work was to investigate the mechanical role of the PTFE felt pad in separating the vessel from the nerve. Therefore, the simulation results are mainly governed by the mechanical properties of the PTFE felt pad material rather than by small variations in the elastic modulus of the nerve tissue. Consequently, this approximation is not expected to significantly influence the main conclusions of the study.

**Figure 4:**
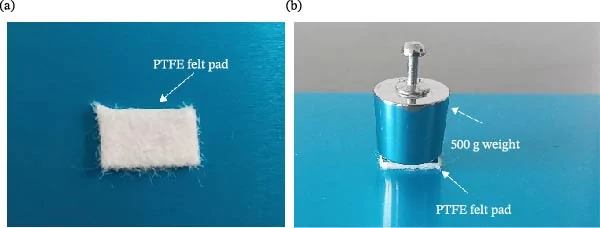
Equivalent testing of compression elastic modulus of the PTFE felt pad: (a) PTFE felt pad and (b) testing of elastic modulus.

Utilize a PTFE felt pad with dimensions of 6.0 mm × 10.0 mm × 1.2 mm as the test sample (see Figure 4a). Weights are applied to the sample to measure the resulting compression deformation, which is used to indirectly determine its compressive elastic modulus (see Figure 4b). When a 500 g weight is applied, the sample compressed to two‐thirds of its original thickness, yielding an estimated compressive elastic modulus of ~0.250 MPa.

**Figure 5:**
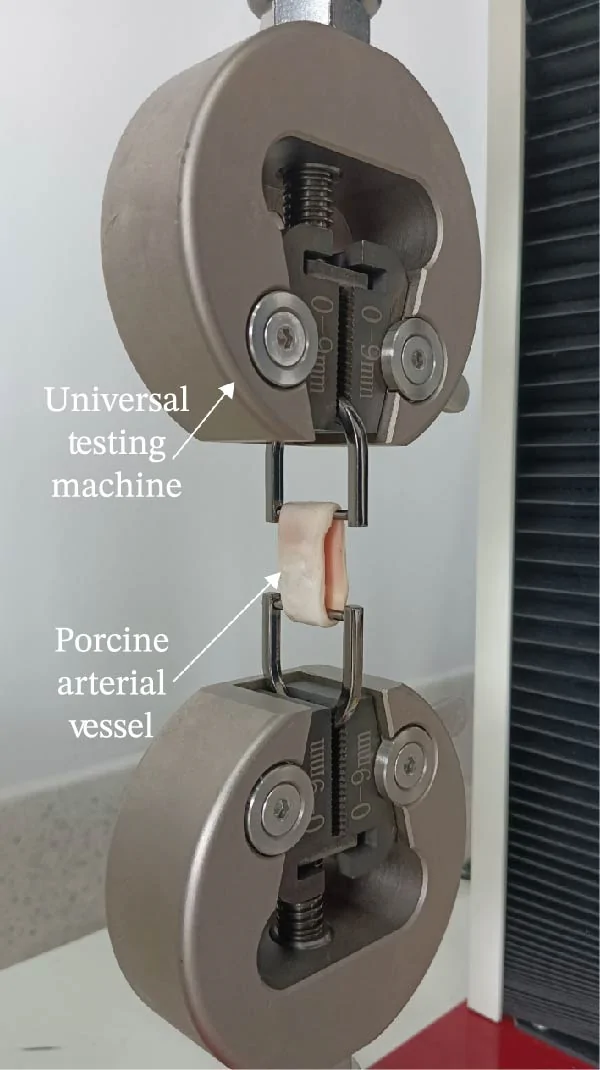
Tensile testing of porcine arterial vessel.

**Figure 6:**
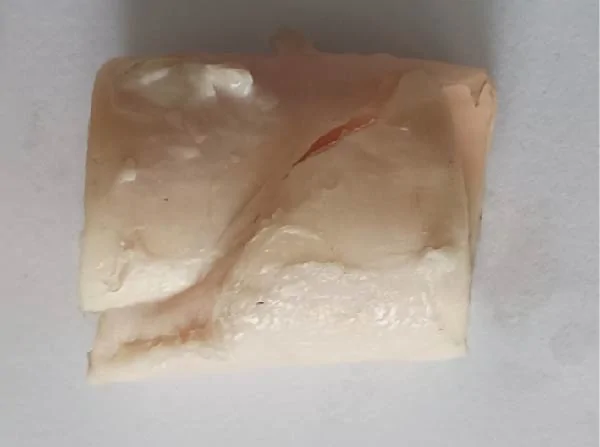
Photograph of the tensile fracture of porcine arterial vessel.

Figure 5 illustrates the setup for in vitro testing of the elastic modulus of blood vessels. The vessel is clamped and secured to the tensile testing machine. A tensile load is applied at a rate of 2 mm/min. As the load progressively increases, the tensile force also escalates until the vessel ruptures, as depicted in Figure 6.

**Figure 7:**
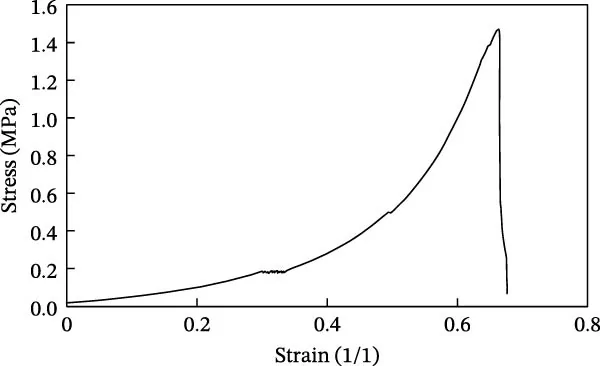
Tensile stress–strain curve of porcine arterial vessel.

The stress–strain data obtained from the tensile test are illustrated in Figure 7, showing a nonlinear relationship where the modulus increases with strain. For simplification in the simulation model, an average value is used as the elastic modulus of the blood vessel. Given that the diameter and thickness of the anterior inferior cerebellar artery near the human trigeminal nerve are smaller than those found in porcine cardiovascular tissues, the overall rigidity is lower. Nonetheless, as the tissue structure is fundamentally similar, the modulus values are considered roughly equivalent, allowing the test results to be applicable to the simulation model. Based on the data, the average estimated modulus of the arterial vessel is determined to be 2.2 MPa, which is also assumed for the modulus of the nerve bundle.

### 3.2. FEM Model Without PTFE Felt Pad

The geometry of the trigeminal nerve and the adjacent vascular structure is constructed based on anatomical data derived from medical imaging, such as MRI or CT scans. The FEM model without MVD is comprised of two primary components: (1) The trigeminal nerve is modeled as a cylindrical structure with a thicker root located near the brainstem. This neural cylinder has a diameter of 3.0 mm and a length of 17.3 mm. (2) The blood vessel, typically represented as the superior cerebellar artery, is modeled as a flexible, tubular structure positioned to simulate compression against the nerve. The outer diameter of the blood vessel is 1.15 mm, and the thickness is 0.23 mm.

**Figure 8:**
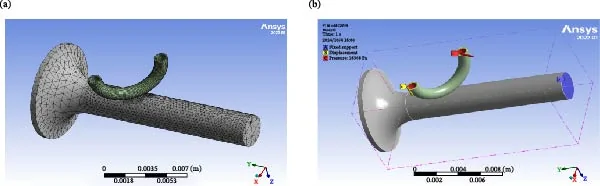
FEM contact model of blood vessels and trigeminal nerve without PTFE felt pad: (a) mesh model and (b) load and boundary conditions.

In ANSYS Workbench, a finite element model was established according to the dimensions shown in Figure 8. In the initial configuration, the blood vessel and the trigeminal nerve were in geometric contact without any compressive load. Frictional contact was defined between the vessel and the nerve with a friction coefficient of 0.1. Fixed boundary conditions were applied to both ends of the trigeminal nerve. A physiological internal pressure of 120 mmHg was applied to the blood vessel, and a displacement load of 1 mm toward the nerve was imposed on the vessel to generate neurovascular compression. The resulting contact stress, deformation, and other mechanical responses were obtained through finite element contact analysis.

The model was discretized using second‐order solid elements. The final mesh consisted of 10,469 elements and 34,770 nodes. To improve the accuracy of the contact analysis, the neurovascular contact region was locally refined to an element size of 0.15 mm. A mesh sensitivity analysis was performed by further refining the mesh, and the variation in the maximum compressive stress of the trigeminal nerve was found to be less than 5%. Therefore, the selected mesh density was considered sufficient to ensure mesh‐independent results.

Prolonged vascular compression can subject the trigeminal nerve to sustained mechanical stress, which is considered to be one of the major factors associated with the development of trigeminal neuralgia. Therefore, the compressive stress acting on the trigeminal nerve was used as the primary biomechanical indicator in the present study.

**Figure 9:**
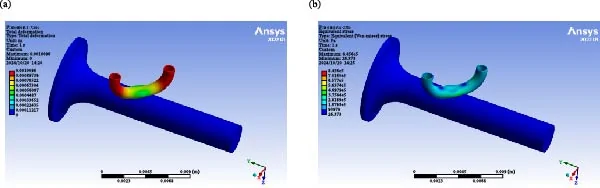
Deformation and stress distribution in the blood vessel and trigeminal nerve without PTFE felt pad by FEM: (a) deformation and (b) stress.

**Figure 10:**
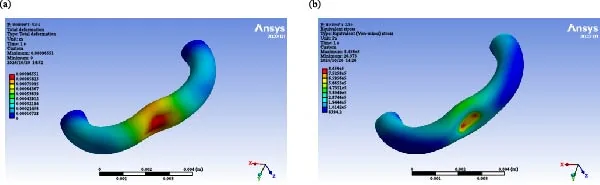
Deformation and stress distribution in the blood vessel without PTFE felt pad by FEM: (a) deformation and (b) stress.

**Figure 11:**
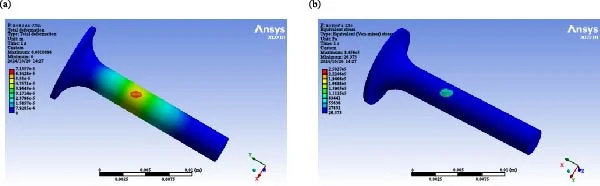
Deformation and stress distribution in the trigeminal nerve without PTFE felt pad by FEM: (a) deformation and (b) stress.

After conducting finite element analysis, the results (shown in Figures 9–11) detail the deformation and stress distribution in blood vessels and the trigeminal nerve without a PTFE felt pad. The maximum compression deformations observed are 0.966 mm in blood vessels and 0.007 mm in nerves. Correspondingly, the maximum stresses recorded are 0.846 MPa in blood vessels and 0.250 MPa in nerves. The constriction of blood vessels as a result of compression subsequently leads to the compression of nearby nerves.

### 3.3. FEM Model With PTFE Felt Pad

**Figure 12:**
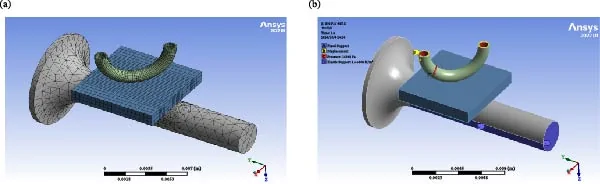
FEM contact model of blood vessels and trigeminal nerve with PTFE felt pad: (a) mesh model and (b) load and boundary conditions.

The FEM model for MVD consists of three parts. The additional third part is the PTFE felt pad: Representing the decompression material used in MVD, it is modeled as a thin elastic layer inserted between the nerve and the blood vessel. The thickness of the PTFE felt pad is 1.2 mm (Figure 12).

**Figure 13:**
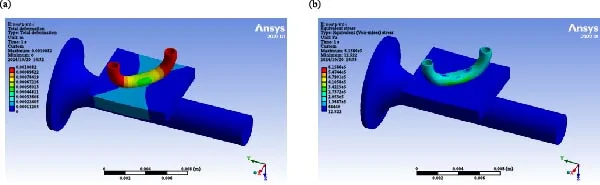
Deformation and stress distribution of blood vessels and trigeminal nerve with PTFE felt pad by FEM: (a) deformation and (b) stress.

**Figure 14:**
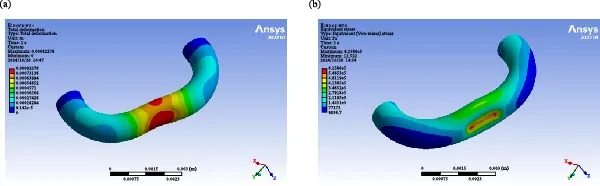
Deformation and stress distribution of blood vessel with PTFE felt pad by FEM: (a) deformation and (b) stress.

**Figure 15:**
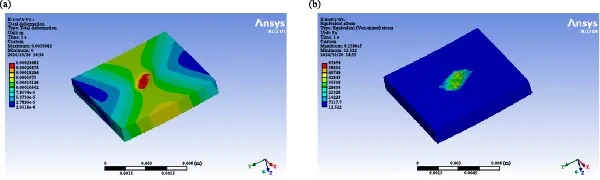
Deformation and stress distribution of PTFE felt pad by FEM: (a) deformation and (b) stress.

**Figure 16:**
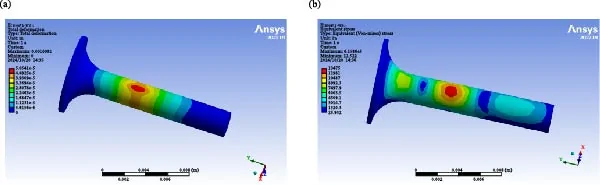
Deformation and stress distribution of trigeminal nerve with PTFE felt pad by FEM: (a) deformation and (b) stress.

The results, shown in Figures 13–16, demonstrate the effectiveness of the FEM with a PTFE felt pad. The maximum compression deformations observed were 0.823 mm in blood vessels, 0.0235 mm in PTFE felt pad, and 0.005 mm in nerves. Correspondingly, the maximum compression stresses recorded were 0.616 MPa in blood vessels, 0.064 MPa in PTFE felt pad, and 0.013 MPa in nerves.

The finite element simulation results (Table 1) illustrate the impact of using a PTFE felt pad. Compared to scenarios without a decompression sheet, the deformation in blood vessels decreased from 0.966 to 0.823 mm, a reduction of 18%, while the deformation in nerves decreased from 0.007 to 0.005 mm, a 29% reduction. Similarly, the stress in blood vessels decreased from 0.846 to 0.616 MPa, a 27% reduction, and the stress in nerves dramatically decreased from 0.250 to 0.013 MPa, corresponding to an ~95% reduction in the present model. The PTFE felt pad substantially reduced the mechanical compression acting on the trigeminal nerve.

**Table 1:** Comparison of structural response with and without PTFE felt pad by FEM.

Structural response parameter	Without PTFE felt pad	With PTFE felt pad	Reduce percentage (%)
Maximum deformation in blood vessel	0.966 mm	0.823 mm	18
Maximum stress in blood vessel	0.846 MPa	0.616 MPa	27
Maximum deformation in trigeminal nerve	0.007 mm	0.005 mm	29
Maximum stress in trigeminal nerve	0.250 MPa	0.013 MPa	95

### 3.4. Discussions

The interaction between the blood vessel and the trigeminal nerve was analyzed using a finite‐element contact model. The simulation reproduced the mechanical compression exerted by the vessel on the nerve and quantified the resulting stress distribution. In the absence of a PTFE felt pad, high compressive stress was concentrated on the neurovascular contact region. Following PTFE felt interposition, the contact pressure was markedly reduced because the pad acted as a compliant buffer between the vessel and the nerve. The finite element results therefore suggest that PTFE felt interposition can effectively reduce local mechanical compression at the neurovascular interface.

#### 3.4.1. Effect of PTFE Felt Pad Modulus

**Figure 17:**
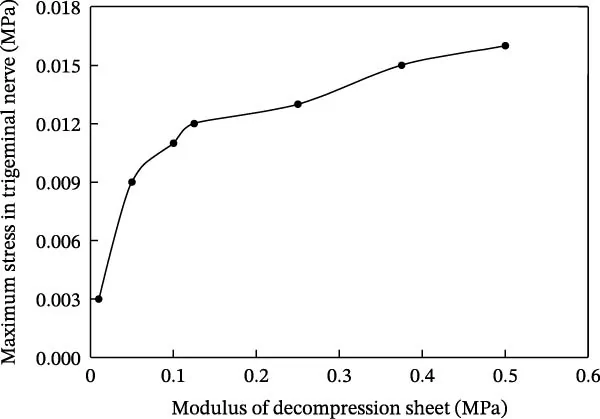
Effect of PTFE felt pad modulus on the maximum compressive stress of the trigeminal nerve by FEM.

The modulus of the PTFE felt pad plays an important role in determining the effectiveness of decompression. As shown in Figure 17, the maximum compressive stress acting on the trigeminal nerve increased with increasing pad modulus. A lower‐modulus material provided greater deformation capacity and improved load redistribution, resulting in lower nerve stress. These results indicate that the mechanical properties of the interposition material can significantly influence the decompression effect.

#### 3.4.2. Sensitivity Analysis of Trigeminal Nerve Modulus

**Figure 18:**
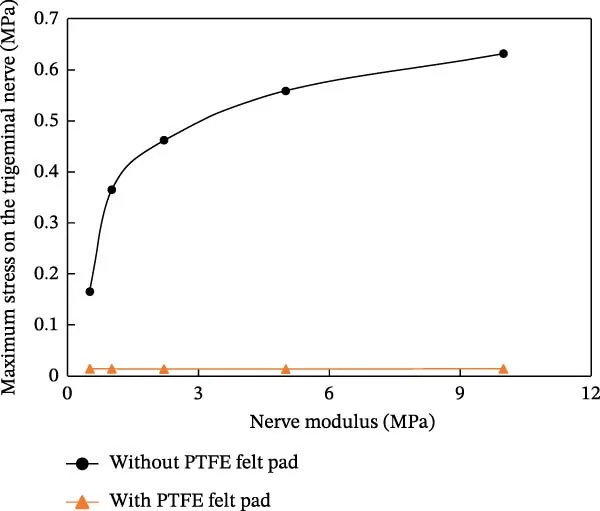
Effect of trigeminal nerve elastic modulus on the maximum compressive stress acting on the trigeminal nerve by FEM.

To evaluate the influence of the assumed nerve material properties, a sensitivity analysis was performed by varying the elastic modulus of the trigeminal nerve from 0.5 to 10 MPa. As shown in Figure 18, the predicted maximum compressive stress increased with increasing nerve modulus in the absence of the PTFE felt pad. However, after insertion of the PTFE felt pad, the compressive stress remained at a consistently low level throughout the investigated modulus range. These results indicate that although the absolute stress values are affected by the assumed nerve modulus, the primary conclusion of the study that PTFE felt interposition substantially reduces local nerve compression remains robust.

#### 3.4.3. Sensitivity Analysis of Friction Coefficient

**Figure 19:**
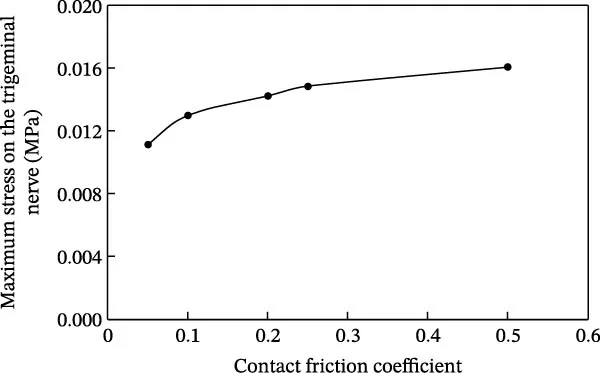
Effect of contact friction coefficient on the maximum compressive stress of the trigeminal nerve by FEM.

A sensitivity analysis was also performed by varying the friction coefficient between the vessel and the trigeminal nerve. As shown in Figure 19, the friction coefficient affected the predicted stress level to some extent, with higher friction coefficients producing slightly higher nerve stresses. Nevertheless, the stress remained at a relatively low level throughout the investigated range, and the overall conclusion that PTFE felt interposition substantially reduces nerve compression was unchanged.

#### 3.4.4. Limitations

Several limitations of the present study should be acknowledged. First, the material properties used in the finite element model were derived from mechanical testing of porcine thoracic aorta tissue. The porcine thoracic aorta was selected because it can be readily obtained for experimental testing. However, it is not mechanically identical to either the intracranial arteries commonly involved in trigeminal neuralgia, such as the superior cerebellar artery, or the trigeminal nerve itself. Therefore, the elastic modulus of the trigeminal nerve was not measured directly but was assumed to be similar to that of the porcine thoracic aorta and was indirectly estimated from the experimentally measured vascular modulus. To evaluate the influence of this assumption, a sensitivity analysis was performed by varying the nerve elastic modulus over a plausible range. Although the absolute stress values changed with the assumed modulus, the qualitative conclusion regarding the substantial reduction in compressive stress following PTFE felt interposition remained unchanged, supporting the robustness of the main findings.

Second, a static finite element model was employed. In clinical reality, trigeminal nerve compression is caused by long‐term pulsatile contact between the offending vessel and the nerve. Therefore, the present model does not fully capture the effects of cyclic loading and chronic mechanical irritation. Investigation of dynamic pulsatile loading will be the subject of future work.

Finally, the present study focused exclusively on the interposition technique, in which a PTFE felt pad is placed between the vessel and the nerve. The findings should therefore not be interpreted as an evaluation of other MVD strategies, such as transposition or combined decompression techniques.

## 4. Conclusion

This study developed a biomechanical model to investigate the contact interaction between a blood vessel and the trigeminal nerve following PTFE felt interposition. Finite element simulations were performed to evaluate the stress distribution and mechanical behavior at the neurovascular contact interface.

The results indicate that PTFE felt interposition substantially reduces local contact pressure and stress concentration at the neurovascular interface. Under the material properties and loading conditions adopted in the present model, the maximum compressive stress acting on the trigeminal nerve was reduced by more than 95%. The PTFE felt pad acts as a cushioning material, attenuating the mechanical load transmitted by vascular compression and reducing deformation in the affected nerve region.

It should be emphasized that the predicted stress reduction is model‐dependent and influenced by the assumed material properties of the trigeminal nerve. Nevertheless, sensitivity analyses demonstrated that the qualitative conclusion of substantial stress reduction remained unchanged over a plausible range of nerve elastic moduli and contact friction coefficients, supporting the robustness of the main findings.

These findings suggest that PTFE felt interposition can effectively reduce local mechanical compression in the specific interposition configuration investigated in this study. However, the present work does not directly assess clinical outcomes, such as postoperative pain relief, recurrence, complications, or quality of life. In addition, the findings are limited to the interposition technique and should not be generalized to other MVD strategies, including transposition or combined procedures. Further biomechanical and clinical studies are required to determine whether the predicted reduction in compressive stress correlates with patient outcomes and long‐term surgical efficacy.

## Funding

This study was funded by the Research Project of Nanjing Drum Tower Hospital (Grant 2025‐B1041).

## Conflicts of Interest

The authors declare no conflicts of interest.

## Data Availability

The data that support the findings of this study are available from the corresponding author upon reasonable request.
